# Insight into Rapid DNA-Specific Identification of Animal Origin Based on FTIR Analysis: A Case Study

**DOI:** 10.3390/molecules23112842

**Published:** 2018-11-01

**Authors:** Yahong Han, Lin Jian, Yumei Yao, Xinlei Wang, Lujia Han, Xian Liu

**Affiliations:** College of Engineering, China Agricultural University, Beijing 100083, China; hyhmelody@cau.edu.cn (Y.H.); jianlin@cau.edu.cn (L.J.); Yaoao@cau.edu.cn (Y.Y.); wangxinlei917@cau.edu.cn (X.W.); hanlj@cau.edu.cn (L.H.)

**Keywords:** DNA, FTIR spectroscopy, rapid identification, PLS-DA, animal origin

## Abstract

In this study, a methodology has been proposed to identify the origin of animal DNA, employing high throughput extension accessory Fourier transform infrared (HT-FTIR) spectroscopy coupled with chemometrics. Important discriminatory characteristics were identified in the FTIR spectral peaks of 51 standard DNA samples (25 from bovine and 26 from fish origins), including 1710, 1659, 1608, 1531, 1404, 1375, 1248, 1091, 1060, and 966 cm^−1^. In particular, the bands at 1708 and 1668 cm^−1^ were higher in fish DNA than in bovine DNA, while the reverse was true for the band at 1530 cm^−1^ was shown the opposite result. It was also found that the PO_2_^−^ V_as_/V_s_ ratio (1238/1094 cm^−1^) was significantly higher (*p* < 0.05) in bovine DNA than in fish DNA. These discriminatory characteristics were further revealed to be closely related to the base content and base sequences of different samples. Multivariate analyses, such as principal component analysis (PCA) and partial least squares-discriminant analysis (PLS-DA) were conducted, and both the sensitivity and specificity values of PLS-DA model were one. This methodology has been further validated by 20 meat tissue samples (4 from bovine, 5 from ovine, 5 from porcine, and 6 from fish origins), and these were successfully differentiated. This case study demonstrated that FTIR spectroscopy coupled with PLS-DA discriminant model could provide a rapid, sensitive, and reliable approach for the identification of DNA of animal origin. This methodology could be widely applied in food, feed, forensic science, and archaeology studies.

## 1. Introduction

Deoxyribonucleic acid (DNA) is the genetic material in all living organisms. Many studies on DNA-specific identification continue to be developed for a range of applications, for example, differentiating murine from mutton meat [1]; identifying poisonous mushrooms [2]; identifying fish species in cooked products [3]; detecting meat and poultry species in ground meats, deli meats, canned meats; and dried meats [4] to ensure food safety. In addition, DNA analysis has also been applied in identifying pork, beef, chicken and mutton origins in food products [5] and feed products [6,7] to control adulteration. Besides, it has contributed to the identification of parasite for the rapid detection of infectious disease [8]. In general, methods for the identification of DNA, such as polymerase chain reaction (PCR), DNA metabarcoding, and polymerase chain reaction-restriction fragment length polymorphism (PCR-RFLP) have been commonly used in these studies. These methods are generally based on specific DNA fragments, and can obtain high accurate rates. However, these methods are time-consuming, expensive, and require skilled labor [9].

Compared with traditional molecular technologies, Fourier transform infrared (FTIR) spectroscopy has the advantage of great simplicity, rapidity and cheap. It is widely employed in the fingerprint identification of molecular composition and structure. Therefore, FTIR spectroscopy could provide a new insight into rapid identification based on the difference in DNA molecular composition and structure. To date, only a few studies have focused on rapid identification using FTIR. For example, it was used for differentiating *japonica* from *indica* rice varieties based on DNA structural differences [10]. Subsequently, this method has been extended to differentiate other species, such as varieties of Chinese cabbage [11], and *Camellia reticulata* Lindl in the Chuxiong population [12]. All these studies deal with plant species. Furthermore, it was used in microorganism identification, such as enterococci [13], and invertebrate animals, such as nematode [14]. However, investigations on the rapid identification of vertebrate animal DNA by FTIR spectroscopy can also be helpful in a range of applications, such as the detection of bovine meat and bone meal in fishmeal, due to that bovine meat and bone meal were prohibited from feeding animals to control bovine spongiform encephalopathy crises in Europe [15], differentiating horse meat from beef to control the “European horsemeat crisis” [5], and the identification of wildlife forensic animal to avoid illegal mammalian wildlife trafficking [16,17]. To the best of our knowledge, the mechanism and model of animal DNA discrimination based on FTIR has not been reported previously.

Based on our previous work [18], high throughput extension accessory FTIR (HT-FTIR) spectroscopy was used in the present study for identification of animal DNA. Bovine and fish standard DNA samples were involved in a case study. The discrimination analysis was developed by a combination of principal component analysis (PCA) and partial least squares-discriminant analysis (PLS-DA). Furthermore, the mechanism of the discrimination was investigated. The methodology reported herein was also validated by use on 20 market meat tissue samples, with emphasis on the analysis of discriminatory characteristics.

## 2. Results and Discussion

### 2.1. Infrared Spectral Characteristics of Bovine and Fish Standard DNA Samples

**Figure 1:**
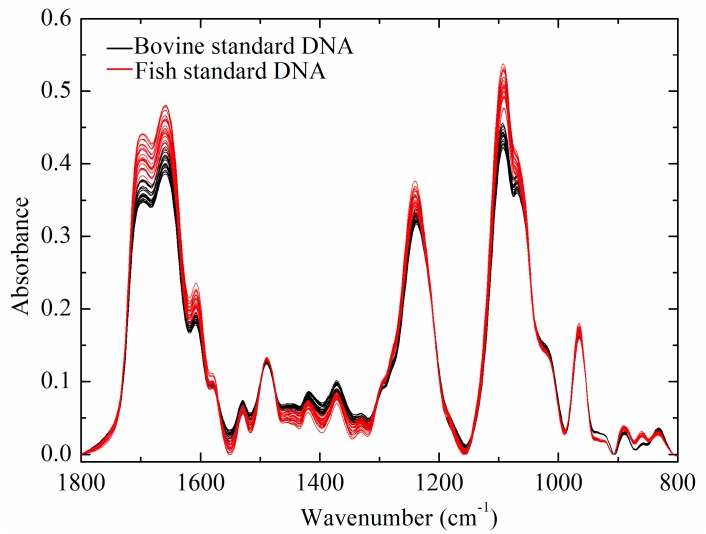
FTIR spectra of calf and salmon DNA.

Figure 1 shows the FTIR spectra of bovine and fish standard DNA samples, which shared similar spectral characteristics. FTIR spectra of DNA samples showed major peaks in three regions, including 1800–1500 cm^−1^, 1500–1250 cm^−1^, and 1250–800 cm^−1^. Similar IR spectral peaks have been identified in related studies [19,20,21,22,23,24]. The assignment of specific absorption bands in three regions are summarized in Table 1. Absorption bands in the region between 1800 and 1500 cm^−1^ were mainly derived from the vibration of double bonds, such as C=N, C=O and C=C [21,25]. These vibrations might be easily sensitive to the effects of base stacking and pairing [21,22]. Absorption intensities in the region from 1500 to 1250 cm^−1^ were mainly attributed to pyrimidine and purine ring modes [19,21,23]. The vibrations of peaks in this region were easily affected by the sugar puckering modes, glycosidic bond rotation, and backbone conformation [21]. Finally, absorption bands in the region (1250–800 cm^−1^) could mostly be attributed to the vibration of PO_2_^−^ groups and deoxyribose stretching, which was extremely sensitive to DNA backbone conformation [11,21,26].

### 2.2. Multivariate Analysis of FTIR Spectral Data

**Figure 2:**
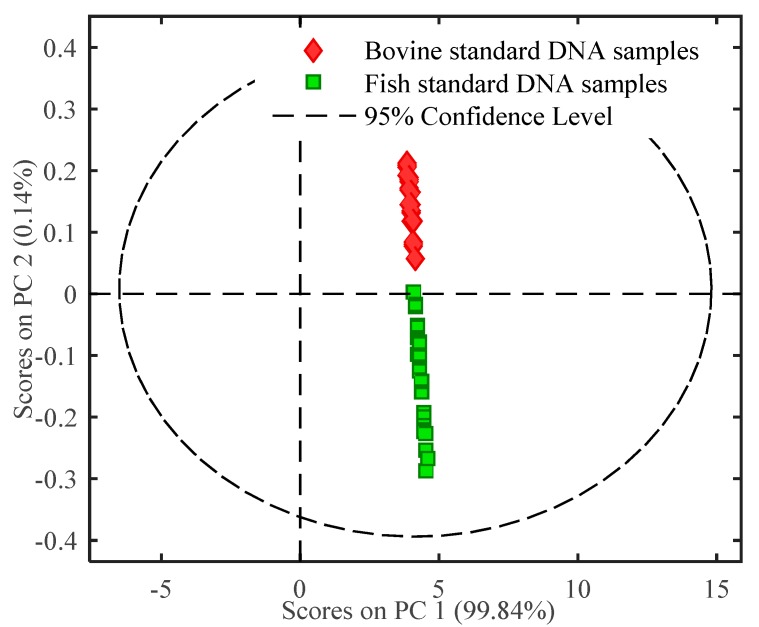
PCA score plot of FTIR spectral characteristics of DNA.

Multivariate analysis was used for further identifying DNA of different animal origins. Initially, PCA was used to assess the separation ability of FTIR spectra of bovine and fish standard DNA samples. The PCA score plot was built using the first two principal components (PCs), as illustrated in Figure 2. PC1 and PC2 could explain most of the variances in the sample clustering, which accounted for 99.84% and 0.14% of the total variation, respectively. As presented in Figure 2, bovine standard DNA showed positive values on PC2, while fish standard DNA samples showed negative scores on PC2. Bovine and fish standard DNA samples were clearly distinguished from each other. It was concluded from PCA result that bovine and fish standard DNA samples were discriminated explicitly without false prediction. Furthermore, a PLS-DA discriminant model was developed based on the DNA characteristics. The calibration model was chosen with leave-one-out cross validation, independent validation was used to assess model accuracy. The PLS-DA model result is shown in Table 2. For all data sets, including the calibration model set, the cross validation set and the independent validation set, both sensitivity and specificity values were 1.00, and the classification error was 0.00. These results indicate that this PLS-DA model was valid and reliable. From the combined results of PCA and PLS-DA, it was concluded that FTIR spectroscopy coupled with chemometrics can be successfully used to differentiate bovine from fish standard DNA samples.

### 2.3. Discussion on the Mechanism of the Discrimination Model

#### 2.3.1. The Average-Difference Profile Analysis

**Figure 3:**
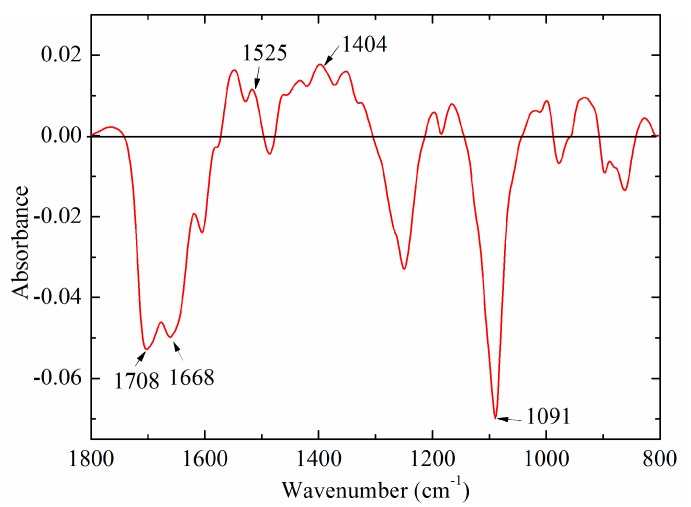
A difference profile between bovine (minuend) and fish (subtrahend) DNA standard samples.

In order to explore the difference between bovine and fish standard DNA samples, the average-difference profile was constructed in Figure 3. The absorption intensities in the region between 1740 and 1600 cm^−1^ were below zero, while the band at around 1530 cm^−1^ was above zero. Both bands at 1708 and 1668 cm^−1^ in the region (1700–1600 cm^−1^) could be due to C=O stretching vibration of thymine, while the band at 1530 cm^−1^ could be associated with the vibration of cytosine and guanine. A similar result was obtained by Mello and Vidal [27], who suggested that FTIR spectra of bovine DNA was higher than that of fish DNA at the band at 1661 cm^−1^, while it showed the opposite result in the 1600–1500 cm^−1^. An important cause may be that bovine DNA has higher GC content, and thus lower AT content, which could have an effect on the vibration of the base pairing, as well as hydrogen bonds [28]. Besides these characteristic peaks, there were other obvious spectral differences at 1404 and 1091 cm^−1^, which may be ascribed to vibration of all bases (adenine, thymine, cytosine, and guanine) and PO_2_^−^ groups [19,24,29].

#### 2.3.2. Comparison of Spectral Characteristics of DNA of Two Groups

**Figure 4:**
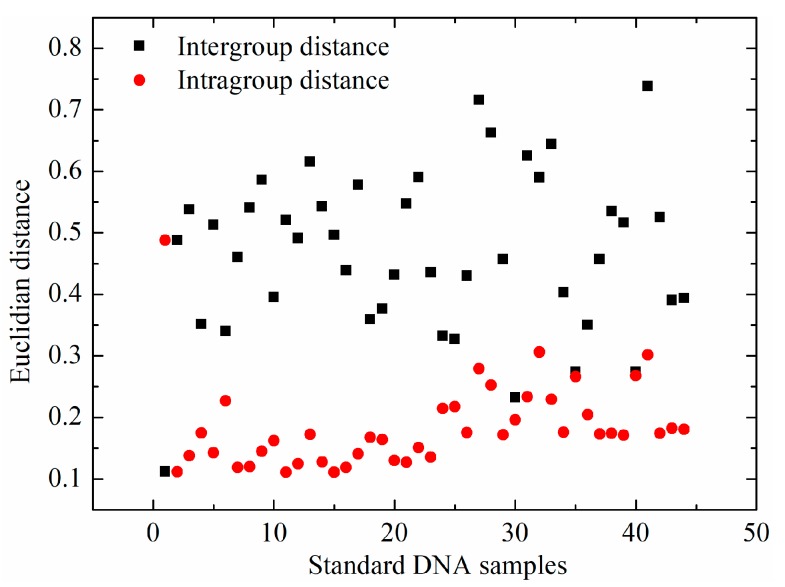
Spectral distance of standard DNA samples including intergroup and intragroup distances.

Besides the analysis of mean spectra of bovine and fish standard DNA samples, spectral distances, including the intergroup and intragroup distances, were analyzed via Euclidian distance. For each standard DNA sample, the intragroup distance was the average of 21 Euclidian distances, which were calculated between one sample and the other within the same type. The intergroup distance was the average of 22 Euclidian distances, between one sample and all the samples in the different group. The intergroup and intragroup distances of each standard DNA sample are illustrated in Figure 4. Average intragroup distance was 0.18 ± 0.06, while intergroup distance between bovine and fish standard DNA samples was 0.48 ± 0.13. More importantly, a nonparametric test of intergroup and intragroup distances was conducted. This revealed that there was a significant difference (*p* < 0.05) between intragroup and intergroup distances, which may explain why bovine and fish standard DNA samples could be differentiated. 

#### 2.3.3. The Contribution of IR Spectral Characteristic Peaks of DNA

**Figure 5:**
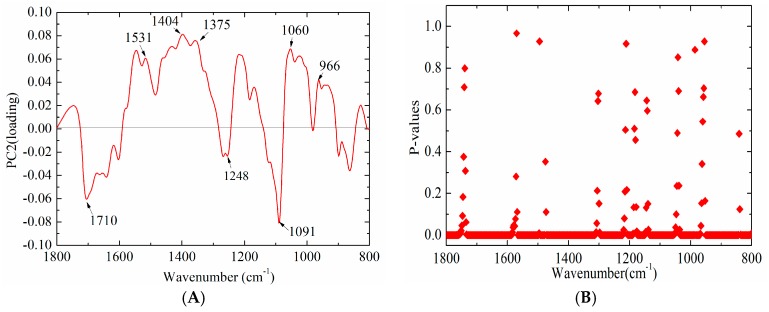
Analysis of the mechanism of the discriminant model ((**A**) loading plot for second principal component (PC2) loading; and (**B**) statistical significance of DNA standard samples in different wavenumbers, based on a *t*-test).

As mentioned above, bovine and fish standard DNA samples could be readily separated by the PCA score plot, especially by PC2 (Figure 2). The PCA loading plot could be used to reveal the most discriminative peaks [30]. Therefore, the loading plot of PC2 was further analyzed for exploring the highest contribution in the discrimination model. As illustrated in Figure 5A, several infrared absorption bands, including 1710, 1531, 1404, 1375, 1248, 1091, and 1060 cm^−1^, could be considered as the highest contribution to the distinction between bovine and fish standard DNA samples. It should be noted that these peaks at 1710, 1531, 1404, and 1091 cm^−1^ were discussed previously (seen in Section 2.3.1). Apart from these bands, the peak at 1375 cm^−1^ was attributed to anti vibration of adenine and guanine [23]. It is consistent with a previous study by Qiu [12], who demonstrated that the band at 1388 cm^−1^ contributed the most to the differentiation among ten varieties of *Camellia reticulata* Lindl. from Chuxiong population. The peak at 1248 cm^−1^ was ascribed to phosphate antisymmetric stretching. Furthermore, it was found that the PO_2_^−^ V_as_/V_s_ ratio of bovine standard DNA samples was 0.75 ± 0.0046, while it was 0.70 ± 0.0048 for fish standard DNA samples. It was further confirmed that the PO_2_^−^ V_as_/V_s_ ratio was significantly higher (*p* < 0.05) in bovine than fish standard DNA samples, which is in close agreement with a previous finding of Mello and Vidal [27]. It could be speculated that the PO_2_^−^ group may play an important role in differentiating two types of DNA. It was also seen that this ratio was different from that reported by Mello and Vidal [27], who suggested that the PO_2_^−^ V_as_/V_s_ ratio of bovine and fish DNA obtained by using ARO objective were 0.92 and 0.87, respectively; while they were 0.67 and 0.61, respectively, when obtained by using ATR objective. The difference may be due to the different sampling techniques used in each case.

To explore the specific contribution of more characteristic peaks in the discriminant model, an independent samples test-analysis of spectral intensities of bovine and fish standard DNA samples was conducted at each spectral wavenumber. Moreover, adjusting *p* values were calculated by false discovery rates to make multiple testing corrections, as shown in Figure 5B. It was found that there were significant differences (*p* < 0.05) in 469 sites of 520 wavenumbers, particularly at 452 sites (*p* < 0.01). This indicated the ability of FTIR spectral analysis for differentiating bovine from fish DNA. It may be speculated that, together, these spectral peaks have an effect on distinguishing bovine from fish DNA samples.

More importantly, these bands were sensitive to base stacking and base pairing, while the alteration of base stacking and pairing could be associated with different base sequences of DNA from different animal origins. Base stacking interactions between pyrimidine and purine bases in the following trend have been demonstrated: Pyrimidine–pyrimidine < purine–pyrimidine < purine–purine [31,32,33]. These interactions could have an influence on absorption intensities of the DNA bands. It has been found that the degree of propeller twist is sensitive to the base sequence of DNA [34].

### 2.4. Methodology Validation of Market Meat Samples

In order to verify the practical applicability of the method developed, genomic DNA of meat tissue samples were used for HT-FTIR spectroscopic analysis.

#### 2.4.1. Bovine and Fish Samples

**Figure 6:**
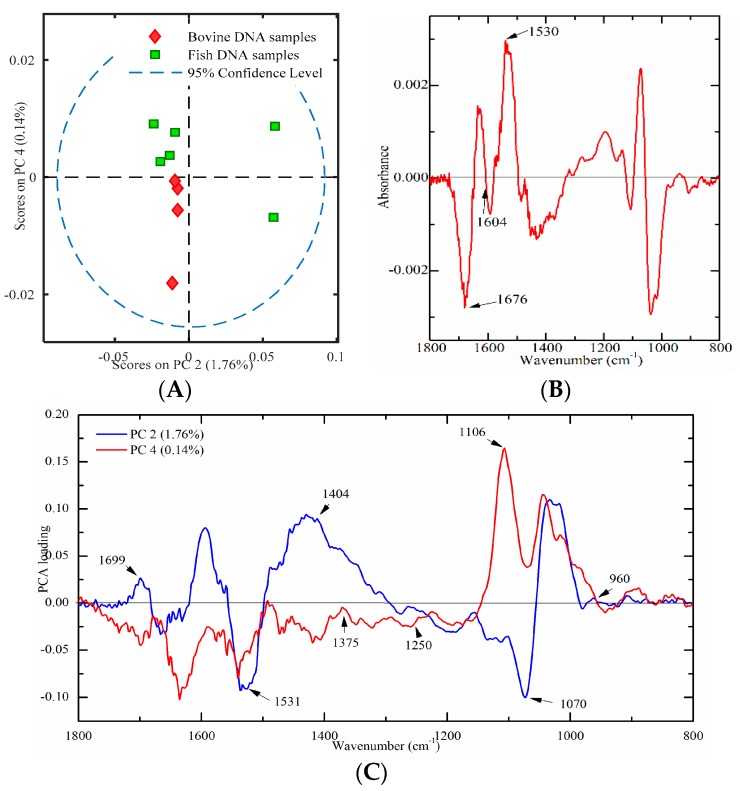
Multivariate analysis of FT-IR spectral data on genomic DNA from meat tissues ((**A**) a PCA plot; (**B**) a difference profile between bovine (minuend) and fish (subtrahend) DNA samples; and (**C**) a PCA loading plot).

Ten meat tissues (four from bovine and six from fish origins) were used to build a PCA score plot to verify this developed method. PC2 and PC4 were used, which could account for 1.76% and 0.14% of the total variation, respectively. As illustrated in Figure 6A, only bovine DNA had negative values onPC2 and PC4, which indicated that bovine and fish DNA samples were successfully differentiated. Furthermore, a PLS-DA model with leave-one-out cross-validation has been developed. Results showed that the sensitivity, specificity and classification error were one, one, and zero, respectively, which indicated that bovine and fish DNA could be successfully separated. In addition, the PO_2_^−^ V_as_/V_s_ ratio was found to be significantly higher (*p* < 0.05) in bovine DNA samples than that in fish DNA samples, which is consistent with results in Section 2.3.3. Thus, it may be concluded that the PO_2_^−^ V_as_/Vs ratio could be an important biomarker for differentiation bovine from fish DNA samples.

In order to explore the contribution of characteristic peaks, the difference profile and the loading plot were analyzed. The difference profile between bovine (minuend) and fish (subtrahend) DNA samples is shown in Figure 6B. Comparison with the results of standard DNA samples (Figure 3), there are several similarities. The peaks at around 1676 cm^−1^ (thymine) and 1604 cm^−1^ (adenine) were below zero, while the band at 1530 cm^−1^ (cytosine and guanine) was above zero (Figure 6B). This phenomenon was consistently observed in Figure 3. The loading plot of PC2 and PC4 is presented in Figure 6C. Several high marked bands, including 1699, 1531, 1404, 1375, 1250, 1106, 1070, and 960 cm^−1^ were identified. All marked bands were in good agreement with Figure 5A. However, compared with Figure 5A, there were several peak shifts, including 1699, 1250, and 1106 cm^−1^. One possible explanation could be that genomic DNA extracted from meat samples was not as pure as standard DNA. Overall, high marked bands, including 1710, 1659, 1608, 1531, 1404, 1375, 1248, 1091, 1060, and 966 cm^−1^, were confirmed in DNA spectra of market meat samples.

#### 2.4.2. Bovine and Porcine Samples

**Figure 7:**
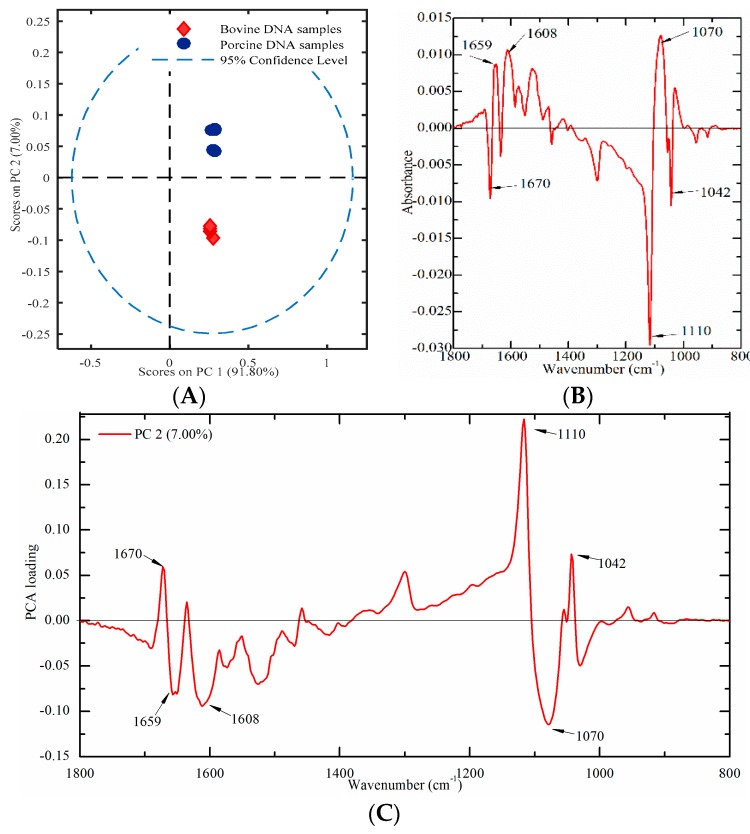
Multivariate analysis of FT-IR spectral data on genomic DNA from meat tissues ((**A**) a PCA plot; (**B**) difference profile between bovine (minuend) and porcine (subtrahend) DNA samples; and (**C**) a PCA loading plot).

Nine meat tissues (four from bovine and five from porcine origins) were used to build a PCA score plot to verify this proposed method. A PCA score plot was established by using PC1 and PC2, which could account for 91.80% and 7.00% of the total variation, respectively. As presented in Figure 7A, porcine DNA had positive values, while bovine DNA samples had negative values on PC2, which indicated that bovine and porcine DNA samples could be clearly separated. In order to explore the contribution of characteristic peaks, the difference profile and the loading plot were also analyzed. The difference profile between bovine (minuend) and porcine (subtrahend) DNA samples and the loading plot of PC2 are presented in Figure 7B,C. Several characteristic peaks at 1670, 1659, 1608, 1110, 1070, and 1042 cm^−1^ were identified.

#### 2.4.3. Bovine and Ovine Samples

**Figure 8:**
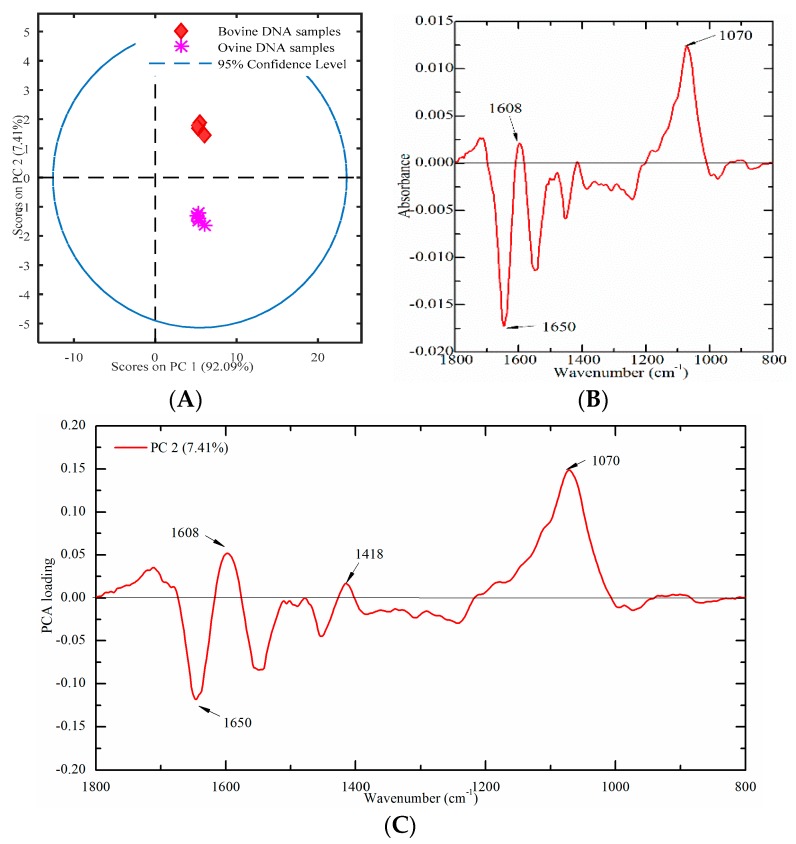
Multivariate analysis of FT-IR spectral data on genomic DNA from meat tissues ((**A**) a PCA plot; (**B**) difference profile between bovine (minuend) and ovine (subtrahend) DNA samples; and (**C**) a PCA loading plot).

Nine meat tissues (four from bovine and five from ovine origins) were used to build a PCA score plot to verify this established method. A PCA score plot was built using PC1 and PC2, which could account for 92.09% and 7.41% of the total variation, respectively. As presented in Figure 8A, ovine DNA had negative values, while bovine DNA samples had positive values on PC2, which indicated that bovine and ovine DNA samples could be clearly separated. In order to explore the contribution of characteristic peaks, the difference profile and the loading plot were also analyzed. The difference profile between bovine (minuend) and ovine (subtrahend) DNA samples are presented in Figure 8B. The most significant spectral variations were a concentrated distribution in the region of 1800–1500 cm^−1^. It may be due to that the characteristics bands in this region, which are sensitive to DNA base pairing, base stacking, and the propeller twist of the DNA structure. Furthermore, the loading plot of PC2 was analyzed in Figure 8C. Several high marked bands, including 1650, 1608, 1418, and 1070 cm^−1^ were revealed.

## 3. Material and Methods

### 3.1. Materials

Double-stranded DNA derived from calf thymus (bovine standard DNA) and salmon testis (fish standard DNA) were purchased from Sigma (Sigma-Aldrich, St. Louis, MO, USA). 20 meat tissue samples (four from bovine, five from ovine, five from porcine, and six from fish origins), were purchased from a local market in Beijing, China. Minced beef tenderloin, porcine leg, ovine leg and fish meat tissues were used in our study. TIANamp genomic DNA kit (DP304, supplied with buffer PW, Rnase A, GD, GB, GA, proteinase K and column CB3) was obtained from Tiangen (Beijing, China). Ethanol (99.9%) was purchased from Beijing Chemical Co., Ltd. (Beijing, China) and nucleasefree water was obtained from Promega (Madison, WI, USA) was used.

### 3.2. Preparation of DNA Samples

Genomic DNA was extracted from 20 meat tissue samples by TIANamp genomic DNA kit following supplier instructions [35]. Briefly, 20 mg of animal tissue were treated to cells suspension, which were then centrifuged (10,000 rpm) for one min. The supernatant was discarded and 200 μL of GA buffer were added to resuspend the cell pellet. Next, 4 μL of RNase A and 20 μL of Proteinase K were added in succession. The solution was then incubated at 56 °C until the tissue is completely lysed; next, 200 μL of GB buffer were mixed with the solution, and this was incubated at 70 °C for 10 min for homogenizing before adding 200 μL of ethanol, mixing and then pipetting into a CB3 spin column. This column was centrifuged at 12,000 rpm for 30 s and then placed into the collection tube. This procedure was repeated three times, with 500 μL of GD buffer, 600 μL of PW buffer and another 600 μL of PW buffer in succession. After this, the CB3 spin column was centrifuged at 12,000 rpm for two min to dry the membrane completely. Finally, 50 μL of water were pipetted to the membrane of the CB3 spin column, which was then incubated at room temperature for two min, and again centrifuged at 12,000 rpm for two min. The solution was then collected into a new, clean 1.5-mL microcentrifuge tube.

In all, 71 DNA samples were involved in this study, including 44 standard DNA samples (25 from bovine and 26 from fish origins) as a calibration set, 7 standard DNA samples (3 from bovine and 4 from fish origins) as an independent validation set, and 20 DNA samples for market meat-tissue validation. The final concentration of all DNA samples was adjusted 100 ng/μL.

DNA sample purity was evaluated by calculating the absorption ratio at 260/280 nm using a micro-UV spectrophotometer (Nanodrop, Thermo Fisher Scientific, San Jose, CA, USA) for further confirmation [36,37,38].

### 3.3. HT-FTIR Measurements

The sampling technique and pretreatment temperature in FTIR spectroscopic analysis of DNA were according to our previous report [18]. Briefly, prior to FTIR measurements, 15 μL of DNA solution were dried on a 96-well silicon plate (Bruker, Rheinstetten, Germany) at 30 °C for 30 min. A Tensor 27 FTIR spectrometer model coupled with a high throughput extension (HTS-XT) accessory (HT-FTIR, Bruker Inc., Germany) was used in all of the measurements. All spectra were collected between 4000 and 400 cm^−1^ with a spectral resolution of 4 cm^−1^. 64 scans were co-added to improve the signal- to-noise ratio [39]. The IR spectra of each sample were run in triplicate.

### 3.4. Data Processing and Multivariate Analysis

The FTIR spectral data in the region (1800−800 cm^−1^) was analyzed using the Matlab version R2015 (Mathworks, Natick, MA, USA). Multiplicative scattering correction (MSC) was used prior to the multivariate analysis, including PCA and PLS-DA [40,41,42]. Preprocessing and multivariate analysis were performed with the PLS Toolbox 8.0 (Eigenvector Research, Wenatchee, WA, USA). Moreover, sensitivity (percentage of correct positive results), specificity (percentage of correct negative results), and classification error (percentage of false results) were used to evaluate the discrimination model [43].

In addition, the mechanism of differentiation between bovine and fish DNA samples was explored by a combination of difference profile analysis, the nonparametric test of spectral distances and the independent samples test analysis of infrared intensities at each wavenumber. The nonparametric test and the independent samples tests were analyzed by software package SPSS 20.0 for Windows (SPSS Inc., Chicago, IL, USA).

## 4. Conclusions

In conclusion, HT-FTIR spectroscopy was demonstrated to be a simple, rapid and sensitivity method to identify genomic DNA from different animal origins. Both DNA standard samples and DNA from meat tissues samples were correctly differentiated. Important discriminatory peaks for bovine/fish model were identified. These peaks were sensitive to base pairing, base stacking, and glycosidic bond rotation, which were closely associated with the base sequence and GC contents. These results, combined with literature analysis, allow us to further speculate that HT-FTIR spectroscopy coupled with PLS-DA discriminant model could identify the DNA of animal origin within different subspecies. This methodology may be used in a wide array of applications, including food adulteration, archaeology, and forensic authentication.

## Figures and Tables

**Table 1:** Assignment of characteristic peaks of bovine and fish standard DNA samples.

Assignment	Comment	Frequencies		References
		Bovine Standard DNA	Fish Standard DNA	
Thymine	C=O stretching	1712	1711	[19,21,23,24]
Thymine	C=O stretching	1659	1659	[19,20,22]
Adenine	Base/in-plane vibration	1608	1608	[20,22]
Cytosine, Guanine	Base/in-plane vibration	1529	1529	[19]
Adenine, Guanine	Ring vibration, C=N	1489	1489	[21]
Adenine, Guanine	Base/in-plane vibration	1420	1420	[19]
Adenine, Guanine	dA, dG anti	1371	1371	[23]
Backbone-A form	V_as_ PO_2_^−^	1238	1240	[22]
Backbone	V_s_ PO_2_^−^	1094	1092	[20]
Backbone	O-P-O bending	964	964	[19]
Deoxyribose	Deoxyribose ring vibration	889	889	[21]
Deoxyribose	Deoxyribose-phosphate, B-marker	831	833	[19]

**Table 2:** Result of PLS-DA discriminant analysis.

	Bovine DNA Standard Samples	Fish DNA Standard Samples
**Sensitivity (Cal)**	1.00	1.00
**Specificity (Cal)**	1.00	1.00
**Classification error (Cal)**	0.00	0.00
**Sensitivity (CV)**	1.00	1.00
**Specificity (CV)**	1.00	1.00
**Classification error (CV)**	0.00	0.00
**Sensitivity (Val)**	1.00	1.00
**Specificity (Val)**	1.00	1.00
**Classification error (Val)**	0.00	0.00

The Cal refers a calibration set; The CV refers a cross-validation set; The Val refers an independent-validation set.
